# Clinical Society of Maryland

**Published:** 1894-01

**Authors:** H. O. Reik

**Affiliations:** 525 N. Howard St.


### CLINICAL SOCIETY OF MARYLAND.

STATED MEETING HELD DECEMBER 1ST, 1893.

The 286th regular meting of the Clinical Society was called to order by the president, Dr. J. Edwin Mitchell.

Dr. Hunter Robb read a paper on

"THE IMPORTANCE OF A BACTERIOLOGICAL TRAINING TO THOSE WHO WISH TO PRACTICE ASEPTIC SURGERY."

Dr. A. K. Bond read a paper entitled

"SOME NERVOUS EFFECTS OF THE DIGESTIVE DISORDERS.

(See page 8.)

DISCUSSION.

Dr. David Street: I have been very much interested in Dr. Bond's paper, but am hardly prepared to agree with him entirely. I can scarcely believe that Diabetes Mellitus can be brought on by indigestion, for in this disease digestion is usually good.

Then too, in the case cited in which the patient had convulsions, possibly if the urine had been examined some other cause might have been discovered. The remedy used would also have relieved an uræmic convulsion, so the diagnosis is incomplete.

Dr. R. B. Norment: I agree with Dr. Bond in believing that nervous affections are frequently due to poison absorbed in the digestive tract. If we have stomach disorders caused by mental diseases, why not nervous disturbance from stomach trouble.

I believe in Uræmia as a cause of convulsions but the simple fact that albumen is found in the urine does not prove the cause in a given case, because of the vast number of cases in which we find albumen but no kidney trouble.

Dr. J. A. Seligman: I have recently seen a case which is called to mind by this paper. A man age 41 complaining of severe headache, pain in the back and constipation. Took a dose of castor oil and was temporarily relieved. The pain and headache returned, however, and were accompanied by tenderness in Right Illiac region and temperature of $102\frac{2}{5}°$. Ordered Quin. Sulph. gr. 5 every four hours. Next day, after having taken 30 grains of quinine temperature was 103°. I administered sponge bath but temperature continued up. During that day some one of his friends suggested a dose of Compound Cathartic pills.

He took them, had fifteen operations during the night, and next morning was free of pain and temperature was $99\frac{2}{5}°$. By night it was normal.

DR. DAVID STREET: We do frequently find albumen in urine for all periods and do not have convulsions, and also do not have Bright's Disease for years without attendant convulsions.

In examining urine I consider the specific gravity of most importance. If I find a low sp. gr. with albumen I fear convulsions, while on the other hand if the sp. gr. is normal the albumen alone causes me no alarm. I have never seen convulsions in an adult due to digestive troubles.

DR. BOND: Dr. Street agrees with me that in children we often have convulsions due to digestive trouble, then why should these influences not be reproduced in adults. The Ptomaines, which are accepted as poisonous of the nervous system, are the result of decomposition, and here is just such a condition to produce them.

In Uræmia we have, we suppose, some substance in the blood which should have been excreted by the kidneys. In my cases I think we had some substance in the blood which should have been carried off by the intestinal tract.

DR. GEORGE A. FLEMING: Will Dr. Bond please state how he decided that to be an epileptic and not an uræmic convulsion ?

DR. BOND: There had been no previous indications of kidney trouble, while there were positive symptoms of intestinal trouble which I considered capable of poisoning the system.

DR. J. H. BRANHAM: In Puerperal convulsions, when death follows, kidney affection has generally been found. I fear we are liable to fix our minds on one thing as the cause of convulsions. Free purgation is a good thing but I think it would be well always to examine the urine.

DR. J. EDWIN MICHAEL: In a matter so grave as convulsions I think you should examine everything about the patient in making diagnosis. As to distinction between Epileptic and Uræmic convulsions, most authorities say they are alike in character but have different causes.

H. O. REIK, M. D.
525 N. Howard St. Secretary.

